# Revision of *Hemiquedius* Casey (Staphylinidae, Staphylininae) and a review of beetles dependent on beavers and muskrats in North America

**DOI:** 10.3897/zookeys.702.19936

**Published:** 2017-09-25

**Authors:** Adam Brunke, Aleš Smetana, Duncan Carruthers-Lay, Joel Buffam

**Affiliations:** 1 Canadian National Collection of Insects, Arachnids and Nematodes, Agriculture and Agri-Food Canada, 960 Carling Avenue, Ottawa, ON K1A 0C6, Canada

**Keywords:** nidicoly, sympatric speciation, cryptic diversity, Nearctic, Staphylinini, Acylophorina

## Abstract

Based on newly discovered characters on the male genitalia, external morphology and an accumulation of ecological data, we revise the single member of the genus *Hemiquedius*. Two new species, *H. infinitus* Brunke & Smetana, **sp. n.** and *H. castoris* Brunke & Smetana, **sp. n.**, from eastern North America are described, and *H. ferox* (LeConte), restricted to peninsular Florida, is re-described. *Hemiquedius castoris* is strongly associated with the microhabitats provided by nest materials of the North American beaver and muskrat. A key to the three species of *Hemiquedius* is provided and diagnostic characters are illustrated. We also review the beetles known to be obligate associates of beavers and muskrats, and discuss the potential role of these keystone vertebrates in beetle evolution and distribution. Based on nest-associated beetles and their closest living relatives, beaver and muskrat lodges may extend distributions northward by moderating winters, promote sympatric speciation and act as refugia against extinction of lineages on a broader timescale. Further research into these potential impacts by ecologists and evolutionary biologists is encouraged.

## Introduction

**Figure 1.**
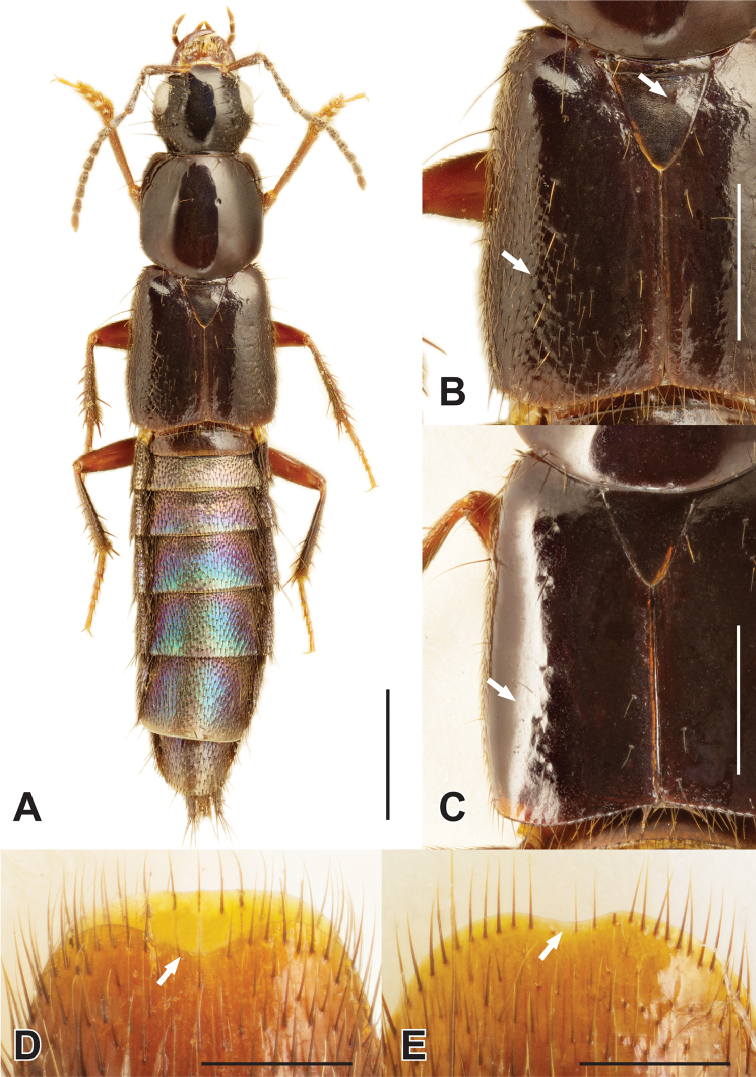
Habitus of *Hemiquedius castoris* Brunke and Smetana (**A**); *H. castoris*, elytra and scutellum (**B**); *H. infinitus* Brunke and Smetana, elytra and scutellum (**C**); *H. ferox* (Casey), male sternite VIII (**D**); *H. castoris*, male sternite VIII (**E**). Scale bars: 2 mm (**A**); 1 mm (**B–E**).

The genus *Hemiquedius* (Fig. 1A) is endemic to North America and a member of the predaceous rove beetle subtribe Acylophorina (Staphylininae: Staphylinini) (Brunke et al. 2016). Currently, one widespread eastern species, *Hemiquedius ferox* (LeConte, 1878), is recognized that occurs from northern Ontario, south to eastern Texas, east to Florida and north to Nova Scotia (Smetana 1971a, 1978, 1990). It inhabits a wide variety of water-soaked decaying organic matter at the edges of forested and open wetlands and is consistently but sparingly collected by submerging this debris by ‘treading’ it under foot (see Smetana 1971a). Several specimens of *Hemiquedius* collected from beaver and muskrat houses were observed by Smetana (1971b) to differ from all others by their elytral setation but were not recognized as a separate species at that time due to limited material and a lack of other corroborating differences. Since then, an entire rove beetle assemblage has been discovered that is strongly associated with the nest materials of beaver and muskrat houses, including several species new to science (Campbell 1979, Hoebeke 1985, Smetana 1995). Most of these species were described relatively recently and were overlooked until beaver and muskrat nests were targeted for sampling.

Here we re-assess the taxonomic status of the ecologically specialized population of *Hemiquedius* and other putative species based on morphological characters and CO1 barcode data. Although ecosystem engineering by beavers is well known to positively impact populations of wetland fauna such as fish, birds, reptiles and invertebrates (Rosell et al. 2005), the promotion of invertebrate speciation by beavers is not well understood. Based on the diversity of lineages that have evolved specialized associations with beaver lodge-building activity, their impact on speciation may be significant and widespread. To facilitate future investigation of this phenomenon, we review the beetle species that appear to specialize on the microhabitat provided by beaver and muskrat houses.

## Material and methods.

### Specimens were examined from the following institutions:

**BIO** Biodiversity Institute of Ontario, Guelph, Ontario, Canada (V. Levesque-Beaudin, J. deWaard)

**CNC** Canadian National Collection of Insects Arachnids and Nematodes, Ontario, Canada

**DEBU** University of Guelph Insect Collection, Ontario, Canada (S. Marshall)

**MEM** Mississippi State University, Mississippi, U.S.A. (via S. Chatzimanolis, Chattanooga, Tennessee)

**FMNH** Field Museum of Natural History, Illinois, U.S.A. (C. Maier, M. Thayer, A. Newton)

**TAMU** Texas A&M University, Texas, U.S.A. (Karen Wright)

### Microscopy, illustration, photography and mapping

All specimens were examined using a Nikon SMZ25 stereomicroscope. To allow for the routine dissection of the terminal abdominal segments (including the aedeagus), distilled water was applied directly to the tip of the abdomen using a fine paintbrush. As a precaution against DNA degradation, specimens examined in the present study were never subjected to high ambient humidity relaxing chambers or entirely submersed in water. Genitalia were washed with 70% alcohol and placed in glycerin for observation. Genitalia were placed in glycerin filled vials for long-term storage, which were pinned with their respective specimen.

Measurements were performed using the live measurement module in NIS Elements BR v4.5. Measurements were taken as listed below, but only proportional (HW/HL, PW/PL, EW/EL, ESut/PL, PW/HW) and forebody measurements were stated directly in descriptions due to variability in body size. Total body length is generally difficult to measure accurately in Staphylinidae due to the contractile nature of the abdomen. Abbreviations for measurements are as follows:

**HL** Head Length, at middle, from the anterior margin of frons to the nuchal ridge.

**HW** Head Width, the greatest width, including the eyes.

**PL** Pronotum Length, at middle.

**PW** Pronotum Width, greatest width.

**EL** Elytral Length, greatest length taken from level of the anterior most large, lateral macroseta to apex of elytra. Its length approximates the length of the elytra not covered by the pronotum and therefore contributing to the forebody length.

**EW** Elytral Width, greatest width.

**ESut** Sutural Length, length of elytral suture.

**Forebody** HL + PL + EL.

Line illustrations were performed in Adobe Illustrator CS6 based on photographs. Photomontage was accomplished using a motorized Nikon SMZ25 microscope and NIS Elements BR v4.5. Photos were processed in Adobe Photoshop CS6. Distribution maps were created using SimpleMappr (Shorthouse 2010). The graph of pronotum length versus width was created in Microsoft Excel and then modified in Adobe Illustrator. Regression lines were added in Excel but for qualitative purposes only.

### Molecular data

Extraction, amplification and sequencing of the barcoding fragment of *CO1* was performed by the Biodiversity Institute of Ontario (**BIO**) (Guelph, Ontario, Canada). Sequences were uploaded to Barcode of Life Datasystems v4 (**BOLD**) (http://www.boldsystems.org) and those sequences deemed to be barcode compliant by BOLD were assigned BINs (Barcode Index Numbers, Ratnasingham and Hebert 2013) as tentative species hypotheses. Using the Taxon-ID tree tool in the workbench of BOLD, barcodes with BINs were visualized in a neighbor-joining tree using the BOLD aligner and Kimura-2 Parameter distances. The barcodes are available in BOLD as the published dataset DS-HEMIQUED. Novel sequences were uploaded to GenBank under the accession numbers: MF966147-MF966149.

## Results

A revision of *Hemiquedius ferox* resulted in the recognition of three species: *H. infinitus* Brunke and Smetana, sp. n. and *H. castoris* Brunke and Smetana, sp. n., from eastern North America and *H. ferox* (LeConte), restricted to peninsular Florida. Although *H. infinitus* and *H. castoris* occur sympatrically, *H. castoris* is strongly associated with the nest material of North American beaver and muskrat, while *H. infinitus* occurs outside of this microhabitat, along the margins of various wetlands. A total of three DNA barcodes >500bp (barcode compliant) and two incomplete barcodes (176 and 306 bp) were generated from available dried specimens. An additional 3 specimens of *H. infinitus* were processed but failed to provide sequences, likely due to inadequate preservation. *Hemiquedius infinitus* and *H. castoris* were represented by 3 and 2 sequences, respectively, and both species were represented by barcodes >500bp that were assigned BINs. Their process IDs are given in the material examined section under the corresponding species. Although two BINs were identified by BOLD (BOLD:ABW6323 and BOLD:ACL9384), sequences did not cluster by the morphology and ecology-based species concepts proposed herein (tree not shown here). However, an OTU (operational taxonomic unit) cluster analysis of both compliant and incomplete barcode fragments in BOLD suggested only a single OTU (average distance = 0.94%, maximum distance = 2.78%). It is likely that, with additional barcode compliant sequences in the future, the two existing BINs will be synonymized into one. Molecular data were unavailable for *H. ferox* as DNA from three dried specimens failed to amplify.

### Key to the species of *Hemiquedius* Casey

**Table:** 

1	Elytral disc with fine setation laterally (Fig. 1B); entire scutellum with coarse and distinctly meshed microsculpture (Fig. 1B); usually collected from the nest wall material of occupied and recently abandoned beaver and muskrat houses, occasionally collected in adjacent wetland microhabitats or in flight traps; aedeagus as in Fig. 2C, D, F	***H. castoris* Brunke and Smetana, sp. n.**
–	Elytral disc without fine setation (Fig. 1C); scutellum usually with only fragments of shallow microsculpture, never entirely covered (single specimen seen with only margins missing microsculpture); collected from a wide variety of wetland edge microhabitats but not known from beaver or muskrat houses; aedeagus as in Fig. 2A, B, E, G	**2**
2	Male sternite VIII with distinct emargination (Fig. 1D); median lobe in parameral view with acute apex (Fig. 2A); paramere barely constricted at base (Fig. 2E); pronotum slightly longer than wide (Fig. 3C); peninsular Florida (Fig. 4)	***H. ferox* (LeConte)**
–	Male sternite VIII without or with slight emargination (Fig. 1E); median lobe with obtuse apex (Fig. 2C); paramere distinctly constricted at base (Fig. 2C, G); pronotum slightly wider than long (Fig. 3B); broadly distributed across eastern North America, not known from peninsular Florida (Fig. 4)	***H. infinitus* Brunke and Smetana, sp. n.**

#### Hemiquedius

Taxon classificationAnimaliaColeopteraStaphylinidae

Casey, 1915

Hemiquedius Casey, 1915: 397, 399; Smetana 1971a (diagnosis, as ‘Quediini’); Chatzimanolis et al. 2010 (as Staphylinini: Quediina); Brunke et al. 2016 (as Staphylinini: Acylophorina)

##### Diagnosis.

*Hemiquedius* can be readily recognized as a member of the subtribe Acylophorina by its elongate, non-lobed and cylindrical mid and hind tarsomeres, and the empodial setae of the hind tarsus, which is distinctly longer than that of the foretarsus. Within the subtribe, *Hemiquedius* has a unique habitus (Fig. 1A) but is also distinguished by a combination of: foretibia without distinct spines; at least sutural half of elytron lacking regular, evenly distributed setae; antennae non-geniculate; pronotum roughly parallel-sided.

#### Hemiquedius ferox

Taxon classificationAnimaliaColeopteraStaphylinidae

(LeConte, 1878)

**Figure 2.**
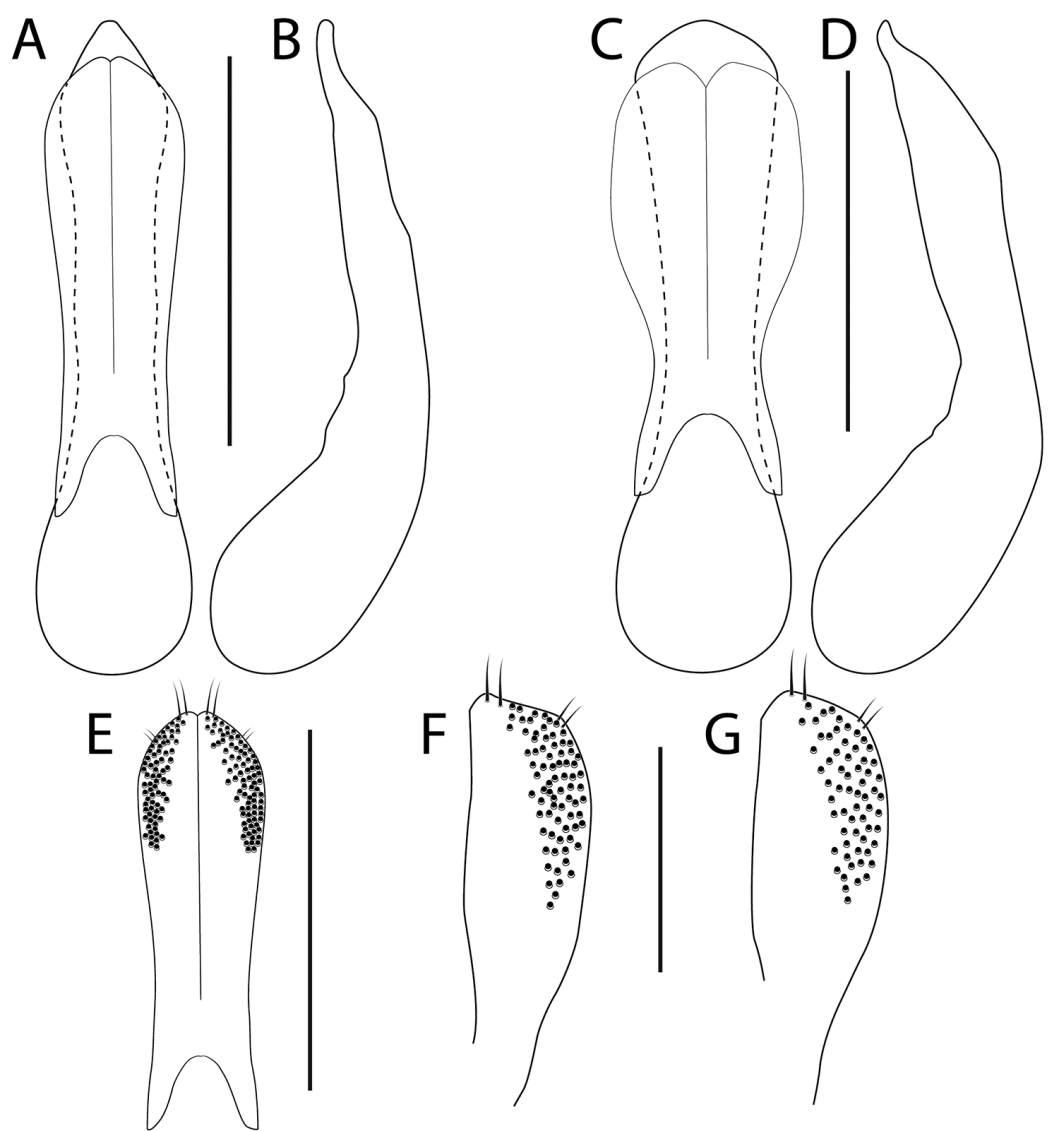
*Hemiquedius ferox* (Casey) (**A, B, E**); *H. infinitus* Brunke and Smetana (**C, D, G**); and *H. castoris* Brunke and Smetana (**F**). Aedeagus in parameral view (**A, C**), median lobe in lateral view (**B, D**), paramere (**E**), left portion of paramere with peg setae (**F, G**). Scale bars: 0.5 mm (**A–E**), 0.25 mm (**F–G**).

**Figure 3.**
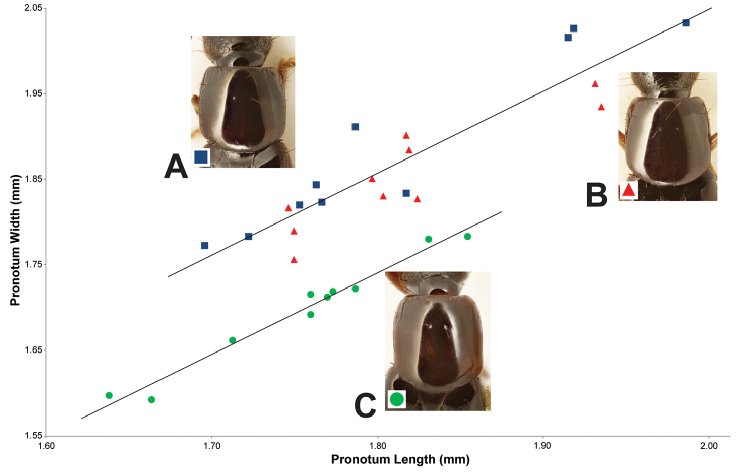
Pronotum shape in *Hemiquedius* species (n = 10, 5 of each sex): *Hemiquedius castoris* Brunke and Smetana **(A)**; *H. infinitus* Brunke and Smetana **(B)** and *H. ferox* (Casey) **(C)**.

**Figure 4.**
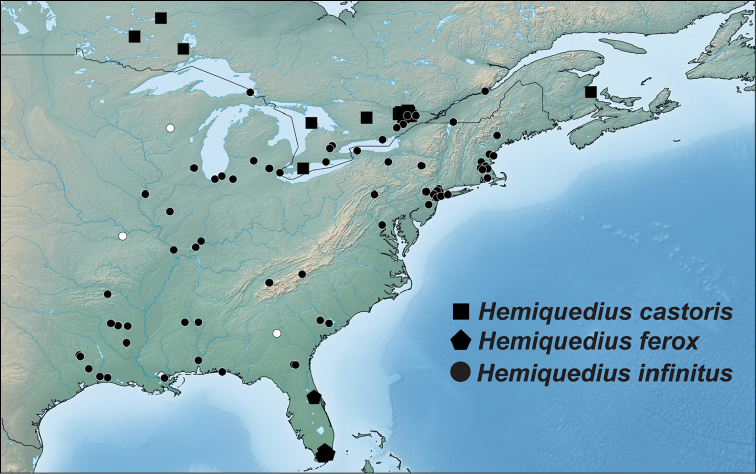
Distribution of *H. castoris* Brunke and Smetana, *Hemiquedius ferox* (Casey), and *H. infinitus* Brunke and Smetana. White markers indicate state only records (placed at the centre of each state) in the absence of more detailed data.

Figs 1D , 2A, B, E , 3C , 4 map

Quedius ferox LeConte, 1878: 388
Hemiquedius ferox : Smetana 1971a

##### Type locality.

Enterprise, Florida

##### Type material.

The male lectotype of *Quedius ferox* designated by Smetana (1971a) is deposited in the MCZ (Museum of Comparative Zoology, Harvard University, Cambridge, United States) and was examined via photographs in the MCZ online type database (http://mczbase.mcz.harvard.edu/name/Quedius%20ferox). Based on the distribution of the lectotype (Enterprise, Florida), pronotum shape and the distinct emargination of male sternite VIII, the name *H. ferox* corresponds to the species restricted to peninsular Florida.

##### Other material.

**UNITED STATES**: Florida: *Dade County*: 50 km SW Miami, Chekika State Rec. area, Grossman Hammock Forest, malaise-FIT, ‘66b’, 15.XI.1985 to 24.II.1986, S. & J. Peck, 1 specimen with CNC656114 BOLD Proc ID CNCCT067-17 (21, CNC); same except 28.VII to 15.XI.1985 (6, CNC); same except 3.III to 28.IV.1985 (2, CNC); same except no date, CNC656113, BOLD Proc ID CNCCT066-17 (2, CNC); Everglades National Park, Mahogany Hammock, forest, UV light, 1.VIII.1981, S. Peck, (1, CNC); Everglades National Park, Long Pine Key, pinelands, malaise-FIT, 8.VI to 26.VIII.1986, S. & J. Peck, CNC656086, BOLD Proc ID CNCCJ3081-14 (1, CNC); Homestead, 28.II.1968, A. Smetana (1, CNC).

##### Diagnosis.

*Hemiquedius ferox* can be distinguished by the distinct emargination of male sternite VIII (Fig. 1D), acute apex of the median lobe (Fig. 2A) and very slightly elongate pronotum (Fig. 3C). It is also the only species known from peninsular Florida.

##### Redescription.

Measurements ♂ (n = 5): HW/HL 1.18–1.20; PW/PL 0.96–0.98; EW/EL 0.91–0.96; ESut/PL 0.69–0.77; PW/HW 1.21–1.25; forebody length 5.03–5.31 mm.

Measurements ♀ (n = 5): HW/HL 1.14–1.18; PW/PL 0.96–0.97; EW/EL 0.92–0.96; ESut/PL 0.72–0.74; PW/HW 1.24–1.28; forebody length 4.80–5.50 mm.

Coloration: body dark brown, pronotum sometimes moderately paler, dark reddish brown, abdomen with strong iridescence; palpi reddish brown; legs except coxae light reddish to reddish brown, paler than rest of body, coxae dark brown; antennae dark reddish brown, apical 1-2 segments slightly paler.

Head transverse, slightly more so in males, temples slightly smaller to slightly longer than eyes, middle of disc without punctures. Antennomeres elongate, antennomere 3 extremely elongate, segments decreasing in length to penultimate, which is slightly elongate.

Pronotum slightly longer than wide, weakly converging anteriad, disc without microsculpture, wider than head (Fig. 3C). Elytra broader and considerably shorter than pronotum at middle, fine, uniform setation restricted to epipleuron (as in Fig. 1C), disc with only a few sparse rows of setae, elytral disc without distinct microsculpture, scutellum at most with shallow fragments of meshed microsculpture, never entirely covered with a coarse mesh.

Abdominal tergites with pubescence moderately sparse, setae separated by far more than their diameter, especially sparse at middle of each disc.

Median lobe in lateral view narrowed to simple apex, which is deflexed ventrad at apical fifth (Fig. 2B); median lobe in parameral view with acute apex, expanded subapically (Fig. 2A); paramere in situ shorter than median lobe (Fig. 2A), broad and scarcely constricted at base, with small median incision, peg setae arranged in a pair of crescent-shaped fields (Fig. 2E); apical margin of male sternite VIII with distinct emargination (Fig. 1D); male tergite X triangular, with apex ranging from obtuse to slightly acute, not emarginate; male sternite IX with small but distinct and semi-circular emargination.

Female tergite X elongate triangular, with thin median extension that gradually extends from lateral margin in most specimens, shape overlapping with some specimens of *H. infinitus*.

##### Distribution.

Figure 4. This species is currently known only from Dade and Volusia counties in peninsular Florida.

##### Bionomics.

Specimens have been collected using FITs in hammock forests, ‘pinelands’, and one specimen came to a UV light. Two specimens were teneral (3.III to 28.IV, 15.XI to 24.II).

##### Comments.

*Hemiquedius ferox* is distinguishable externally from other species of the genus by the slightly longer pronotum and the distinctly emarginate male sternite VIII.

#### Hemiquedius castoris

Taxon classificationAnimaliaColeopteraStaphylinidae

Brunke & Smetana sp. n.

http://zoobank.org/4CE9BE4C-693B-4077-93D6-A61C39726351

Figs 1A, B, E , 2F , 3A , 4 map

##### BINs (shared with *H. infinitus*).

BOLD:ACL9384 + BOLD:ABW6323

##### Type locality.

Gatineau Park, Outaouais region, Quebec, Canada.

**Type material. Holotype** (♂, CNC). “hutte à castor” [=beaver lodge] [small label] / Lac Fortune, Parc Gatineau, Qué, 15.VIII.1976, R. Sexton / CNC Coleo DNA Barcode voucher 00251682 / Barcode of Life, DNA voucher specimen, Sample ID CNC COLEO 00251682, BOLD Proc. ID: CNCCJ3033-14 [blue label].

##### Paratypes.

(19 ♂ 17 ♀ CNC, 22♂ 19 ♀ DEBU): **CANADA: New Brunswick**: *Kent Co.*: Kouchibouguac National Park, 19.XI.1977, Campbell and Smetana, (5, CNC); same except S.J. Miller (1, CNC). **Ontario**: *Bruce Co.*: Stokes Bay, beaver lodge, 1.VI.2008, S.A. Marshall (1, DEBU); *Chatham-Kent Co.*: Rondeau Prov. Park, Marsh Trail, ex. muskrat nest in marsh, 4.VI.1985, A. Davies and J.M. Campbell, specimen CNC656084 with BOLD Proc ID CNCCJ3032-14 (8, CNC); *Kenora Distr.*: 4 mi E. Alcona, 18.VI.1973, Campbell and R. Perry (2, CNC); Ignace, 16.VI.1973, Campbell and Perry (1, CNC); 47 mi S. Pickle Lake, 22.VI.1979, Campbell and Perry (1, CNC); *Ottawa Reg.*, South March, ex. muskrat, 3.V.1969, A. Smetana, BOLD Proc ID CNCCJ3034-14 (1, CNC); Ottawa, Mer Bleue, 3.VII.1973, Smetana and Davies (1, CNC); *Thunder Bay Distr.*: 52 mi N Hurkett, Black Sturgeon Lake, 28.VI.1973, R. Perry and J. M. Campbell (1, CNC). **Quebec**: *Outaouais Reg.*: Gatineau Park, Lac Fortune, hutte à castor, 26.VIII.1976, R. Sexton (1, CNC, 5 DEBU); same except 27.VIII.1976 (1, DEBU); same except 21.VIII.1976 (5, DEBU); same except 29.VIII.1976 (1, CNC); same except 15.VIII.1976 (4, DEBU); same except 6.IX.1976 (1, DEBU) Gatineau Park, Blind Lake, 8.VIII.1969, J. M. Campbell (7, CNC); same except 11.XI.1970, J.M. Campbell and S. Peck (2, CNC); Gatineau, Old Chelsea, hutte de castor, 4.IX.1976, J.F. Laundry (3, CNC); Gatineau Park, Old Chelsea, hutte castor, 26.VIII.1975, R. Sexton (1, DEBU); Gatineau Park, Hay Lake, ex. beaver lodge, 2.X.1976, Campbell and Sexton (1, CNC); Gatineau Park, Lac Hay, hutte à castor, 2.X.1976, R. Sexton (3, DEBU); Gatineau Park, Meech Lake, 8.XI.1967, (1, CNC); Gatineau Park, Lac Kidder, hutte à castor, 10.X.1976, R. Sexton (2, DEBU); Perkins, hutte à castor, 25.VIII.1976, R. Sexton (4, DEBU); Gatineau Park, Lac Holly, hutte à castor, 11.IX.1976, R. Sexton (3, DEBU); Val-des-Monts, Lac Clermont, hutte à castor, 26.VI.1976, R. Sexton (4, DEBU); same except 1.VII.1976 (1, DEBU); same except 28.VI.1976 (1, DEBU); same except 12.VI.1976 (3, DEBU); Quyon, Pontiac, hutte à castor, 5.IX.1976, R. Sexton (3, DEBU); St. Pierre de Wakefield, hutte castor, 11.XI.1975, R. Sexton (1, DEBU).

##### Diagnosis.

*Hemiquedius castoris* can be easily distinguished by the setose lateral portions of the elytral disc (Fig. 1B) and the coarse meshed microsculpture on the entire surface of the scutellum (Fig. 1B). *Hemiquedius infinitus* and *H. castoris* cannot be distinguished by their CO1 barcodes.

##### Description.

Measurements ♂ (n = 5): HW/HL 1.11–1.16; PW/PL 1.01–1.07; EW/EL 0.99–1.04; ESut/PL 0.71–0.77; PW/HW 1.21–1.26; forebody length 5.05–5.81 mm.

Measurements ♀ (n = 5): HW/HL 1.18–1.20; PW/PL 1.02–1.06; EW/EL 0.94–0.97; ESut/PL 0.74–0.77; PW/HW 1.23–1.26; forebody length 5.21–6.06 mm.

Similar to *Hemiquedius ferox* and differing only in the following: palpi and antennae slightly darker, dark brown to dark reddish brown; antennae on average slightly thinner and shorter in appearance, in most specimens antennomere 8 and 9 slightly less elongate; head slightly more transverse in females rather than males; pronotum slightly wider than long; elytral disc with dense fine setae on lateral portion, scutellum with distinct, transverse meshed microsculpture on entire surface; punctures on abdominal tergites slightly denser; median lobe narrowed to shorter apex, length and shape of narrow apical portion highly variable (as in Fig. 2D); median lobe in parameral view with obtuse apex (as in Fig. 2C); paramere more strongly constricted at base (as in Fig. 2C); paired lobe of paramere elongate relative to that of *H. infinitus* (Fig. 2F); apical margin of male sternite VIII without or with barely discernable emargination (Fig. 1E); female tergite X in most specimens with median extension broader at base and more strongly constricted from lateral margin.

##### Etymology.

We describe this species in honor of Canada on its 150^th^ birthday. Like its national animal, the North American Beaver, Canada promotes a diverse community within its greater environment. The species epithet refers to the close association of this rove beetle with beaver lodges.

##### Distribution.

Figure 4. This species is currently known only in Canada from northern Ontario to New Brunswick but very likely occurs broadly across eastern North America where beavers and muskrats occur. Its distribution includes both the boreal and deciduous forest regions.

##### Bionomics.

All specimens with collecting data have been taken from the nest material within beaver or muskrat lodges, some of which were abandoned for several years. Teneral specimens have been collected in August and September.

##### Comments.

*Hemiquedius castoris* is most similar to the sympatric *H. infinitus* but can be easily distinguished based on the fine setation on the elytral disc. The genitalia of these two species are extremely similar and only differ by the shape of the paramere (Fig. 2F vs. 2G). It is interesting to note that all boreal records of *Hemiquedius* represent *H. castoris*. *Hemiquedius castoris* is not likely to be sympatric with *H. ferox* as its host, the North American Beaver, does not occur naturally in peninsular Florida (Peck 2007).

#### Hemiquedius infinitus

Taxon classificationAnimaliaColeopteraStaphylinidae

Brunke & Smetana sp. n.

http://zoobank.org/AC0C138A-ED2B-45D7-9C56-4505DDD5030C

Figs 1C , 2C,D,G , 3B , 4 map

##### BINs (shared with *H. castoris*).

BOLD:ACL9384 + BOLD:ABW6323

##### Type Locality.

3 mi northwest of Orange, Orange County, Texas.

##### Type material.

**Holotype** (♂, CNC). Tex. Orange Co., 3 mi NW Orange, 17.IV.1976, A. Smetana [dissected parts mounted on card].

##### Paratypes.

(13 ♂ 17 ♀ CNC, 1 ♂ 4 ♀ DEBU, 1 sex? BIO, 1 ♀ TAMU, 1 ♀ MEM, 6 ♂ 7 ♀ FMNH): **CANADA: Ontario**: *Haldimand-Norfolk Region*: ~6 km W of St. Williams, Cronmiller prop., 42°40'21"N, 80°29'26"W, forest pools, 5.VII.2011, A. Brunke, debu01149192, debu01146692 (2, DEBU); Backus Woods, north block, treading vernal pools, 42°40'7"N, 80°29'34"W, 23.IV.2011, Brunke and Marshall, debu00340010 (1, DEBU); Long Point, woodlot jct Hwys 24 & 59, leaf litter at very edge of temp pond, 21.IV.1973, H. Frania (1, CNC); *Lanark County*: 13 km S of Smith’s Falls, 17.V.1981, J. M. Campbell (1, CNC); *Leeds and Grenville County*: 4 km N of Westport, 26.V.1981, A. Davies (1, CNC); *Ottawa Region*: Ottawa (1, CNC); 8 km N Limoges, Larose Forest, 45.388°N 75.228°W tread marshy vegetation around ponds, 81 m, 23.VI.2016, A. Brunke and A. Davies (1, CNC). *Prince Edward Co.*: Brimley, 28.IV.1946 (2, CNC); same except 8.V.1946 (1, CNC); same except 9.V.1943 (1, CNC); same except 12.V.1954 (1, CNC); same except 29.IX.1943 (1, CNC); same except 7.V.1933 (1, CNC); *Wellington Co.*: Conc. 11 and Hume Rd, 43.537 -80.134, 27.X.2010, P. Hebert, sample ID BIOUG01310-E07 (1, BIO); Guelph, 4.VI.1985, B. Longpre, (1, DEBU); Guelph, University Arboretum, cedar swamp, 18.IV.2009, A. Brunke, debu00305699 (1, DEBU); Rockwood (1, CNC). **Quebec**: *Communauté*-*Urbaine-de-Quebec*: Quebec, 2.X.1969, C. Chantal (1, FMNH); *Montérégie Reg.*: Philipsburg, 19.IX to 12.X.1972, Dondale and Redner (1, CNC).

**United States: Alabama**: *Mobile County*: Mt. Vernon, 20.III.1932, H. Dietrich (1, CNC). **Arkansas**: *Union County*: Backwaters of Grand Marais Lake, under debris, 9.VII.1974, R. G. Chenowith, CNC656112, BOLD Proc ID CNCCT065-17 (1 CNC), 10 mi S El Dorado, Little Cornie Bayou, treading, 20.V.1974 (1, FMNH). **Florida**: *Okaloosa County*: Fort Walton Beach, 18.III.1976, E.J. Kiteley (1, CNC). **Illinois**: *McHenry County*: Moraine Hills State Park, litter at log, 7.IV.1984, L.E. Watrous (1, FMNH); *Union County*: 1 mi E of Wolf Lake, 8.V.1976, A. Smetana, CNC656110, BOLD Proc ID CNCCT069-17 (2, CNC). **Indiana**: *Porter County*: Tremont, 14.IV.1938 (1, FMNH). **Louisiana**: state record only (1, FMNH). **Massachusetts**: *Middlesex County*: Sherborn, 26.VIII.1934, C.A. Frost (1, CNC); same except 16.X.1881 (1, CNC). **Michigan**: *Berrien County*: Warren Woods, Lakeside, 6.V.1969, W. Suter (1, FMNH). **Mississippi**: *Oktibbeha County*: Noxubee N.W. Refuge, 33.290 -88757, alpha-pinine baited Lindgren funnel, bottomland hardwood forest, 21-29.VII.2009, J.G. Hill & J. Seltzer (1, MEM). **New Jersey**: *Bergen County*: River Vale, 25.V.1980, P.J.D (1, CNC); *Sussex County*: Hopatcong (1, CNC); state record only, Schwarz (1, FMNH). **New York**: *Long Island*: Queens, 18.X.1924, F.M. Schott (1, CNC); *Richmond County*: Staten Island, Concord (1, CNC); *Seneca County*: Willard, V.1970, R. Lenczy (1, FMNH); *Westchester County*: Yonkers, 29.III.1941 (1, FMNH). **South Carolina**: *Dorchester County*: Francis Beidler Forest, 10 km NE Harleyville, bald cypress swamp, FIT, 1-9.V.1987, CNC656111, BOLD Proc ID CNCCT068-17 (1, CNC); *Orangeburg County*: Orangeburg, The Methodist Oaks, 33.419 -80.858, Berlese wet leaf litter in wetland, 27.III.2010, J. & S. Cornell (4, FMNH). **Texas**: *Houston County*: 11 mi E Ratcliff, 24.IV.1976, A. Smetana, CNC656109, BOLD Proc ID CNCCT064-17 (2, CNC); *Orange County*: 3 mi NW Orange, 17.IV.1976, A. Smetana, CNC656108, BOLD Proc ID CNCCT063-17 (1, CNC); *Trinity County*: 12 mi SW Lufkin, 22.IV.1976, A. Smetana (1, CNC); *Tyler Co.*: Kirby State Forest, 30.575 -94.417, ground level FIT, 9-30.III.2003, E. Riley (1, TAMU).

##### Diagnosis.

*Hemiquedius infinitus* can be distinguished by a combination of the elytral disc without fine dense setae (Fig. 1C), pronotum with PW/PL ≥ 1.00 (Fig. 3B) and the indistinct or absent emargination of male sternite VIII (as in Fig. 1E). *Hemiquedius infinitus* and *H. castoris* cannot be distinguished by their CO1 barcodes.

##### Description.

Measurements ♂ (n = 5): HW/HL 1.15–1.21; PW/PL 1.00–1.05; EW/EL 0.92–0.95; ESut/PL 0.72–0.79; PW/HW 1.19–1.27; forebody length 5.24–5.53 mm.

Measurements ♀ (n = 5): HW/HL 1.17–1.21; PW/PL 1.00–1.04; EW/EL 0.91–0.94; ESut/PL 0.75–0.77; PW/HW 1.21–1.27; forebody length 5.30–5.87 mm.

Extremely similar to *H. castoris* and differing only in the following: elytral disc without fine dense setae (Fig. 1C); scutellum with shallow microsculpture in small fragments or with, at most, entire disc except margins with shallow meshed microsculpture; head not sexually dimorphic; pronotum slightly more elongate on average (Fig. 3B); paramere more strongly constricted at base forming a more rounded lobe (Fig. 2G).

##### Etymology.

The species epithet means unbounded in Latin and refers to the occurrence of this species in a variety of wetland habitats, though not inside the lodges of beavers or muskrats.

##### Distribution.

Figure 4. This species is currently known from a wide area of eastern North America: southern Ontario and Quebec, south to the Florida panhandle, west to Texas and north to northern Michigan. Its distribution corresponds well with that of the eastern deciduous forest. All data from Smetana (1971a) can technically be associated with this species except two specimens from peninsular Florida and two from Quebec, with setose elytra.

##### Bionomics.

Specimens have been collected from a variety of wetland edge habitats ranging from open eutrophic ponds to shaded vernal forest pools.

##### Comments.

*Hemiquedius infinitus* is most similar to *H. castoris* but can be easily distinguished based on the lack of fine dense setae on the elytral disc. The genitalia of these two species are extremely similar, variable and only differ by the shape of the paramere. At present *H. infinitus* is not known to be sympatric with *H. ferox* but may overlap with it in northern Florida.

## Discussion

### Species and distributional limits in *Hemiquedius*

A taxonomic revision of *Hemiquedius ferox* revealed the existence of three distinct species: one known only from peninsular Florida and a pair of highly similar and sympatric eastern species that are thus far allopatric with the former. The eastern pair of species represent an unusual situation in Staphylinidae, where CO1 barcodes are not diagnostic and male genitalia are almost identical (*contra* Hendrich et al. 2014, von Beeren et al. 2016). However, non-overlapping differences in external morphology and ecology indicate that these two species are, possibly rather recently, reproductively isolated. Other examples of similar species that are not distinguishable by CO1 barcodes exist in the Coleoptera and an example was discovered by Grebennikov et al. (2017), who explored the longhorn beetle (Cerambycidae) fauna of the Russian Far East. Two species, separable by external morphology and host plant received the same BIN and were not reciprocally monophyletic in neighbor-joining trees (Grebennikov et al. 2017). An almost identical situation to *Hemiquedius* is known in the ground beetles (Carabidae) with *Pterostichus castor* Goulet & Bousquet, strictly associated with beaver lodges, and *Pterostichus ebeninus* Dejean occurring in various forested and unforested wetlands (Larochelle and Lariviere 2003). The species are at least partly sympatric in the northern U.S. and southern Ontario (Bousquet 2012). Male genitalia in these species are identical but there are consistent differences in external morphology, such as presence/absence of an iridescent sheen and shape of the pronotum (Goulet and Bousquet 1983). Copulation in *Pterostichus castor* was observed on the undersides of branches composing the surface of the lodge (Webster and DeMerchant 2012a). A preference for mating in or on the lodge surface may provide a mechanism for reproductive isolation and subsequent speciation in beaver-associated beetles.

The distribution of *Hemiquedius castoris* is certainly greater than that reported here. We expect this species to occur in at least Wisconsin to New England and across the maritime provinces of Canada. Beaver and muskrat lodges are poorly sampled and the presence of *H. castoris* in the boreal forest region makes it likely to be rather widely distributed across Canada. The North American Beaver and Muskrat do not occur in peninsular Florida (Banfield 1974) and *H. castoris* may be truly allopatric with *H. ferox*.

### Obligate associations with beavers and muskrats in the Coleoptera

Thus far, eleven species of beetle are obligate associates of beaver and muskrat lodges in North America (Table 1). Representatives of the Carabidae, Leiodidae and Staphylinidae are known but the evolution of this association has occurred most frequently in the staphylinids. Most of these associates are predators within the lodge but the most extreme of them, the leiodids, are strongly modified for life as adult and larval ectoparasites of beavers, feeding on dead skin and wound exudates (as reviewed in Peck 2006, 2007). Beaver and muskrat lodges represent a stable and predictable microhabitat that is maintained year-round, and therefore are host to a diverse assemblage of obligate and, even more numerous, facultative associates among the beetles (*e.g.*, Bousquet 1985, Smetana 1995, Webster and DeMerchant 2012b). Unlike the other species of the genus and nearly all other Acylophorina, *H. castoris* is distributed north to the boreal forest of northwestern Ontario, possibly due to milder overwintering conditions within its host’s lodge. A similar situation is known for the carabid *Atranus pubescens* (Dejean), which is strongly associated with beaver lodges in the northern part of its range but occurs in caves and deep litter in the southern United States (Bousquet 1985). In North America, the Holarctic rove beetle genus *Coprophilus* is represented by the non-native (western Palaearctic) *C. striatulus* (Fabricius), *C. sexualis* Leech, limited to a small range in the Pacific Northwest, and the obligate beaver-associate *Coprophilus castoris* Campbell in the northeast (Campbell 1979, Hoebeke 1995). *Coprophilus castoris* appears to be much more closely related to the Palaearctic species rather than the other native Canadian species and may be a relict in North America, surviving in a specialized and moderated niche. Therefore, another potential impact of beavers and muskrat lodges on biodiversity may be in the form of a stable refugium against extinction on a broader time scale. Although beavers and muskrats undoubtedly have a positive impact on invertebrate diversity at multiple spatial and taxonomic levels, the mechanisms involved in these interactions remain unexplored and untested by ecologists and evolutionary biologists. We hope that this review draws attention to this interesting phenomenon and the diverse community supported by the beaver, a charismatic national symbol of Canada.

**Table 1.** Obligate associations of beetles with the lodges of beavers and muskrats in North America.

Family	Subfamily	Species
Carabidae	Harpalinae	*Pterostichus castor* Goulet & Bousquet (Goulet and Bousquet 1983)
	Patrobinae	*Platypatrobus lacustris* Darlington (Larochelle and Lariviere 2003)
Leiodidae	Platypsyllinae	*Leptinillus validus* (Horn) (Peck 2007)
		*Platypsyllus castoris* Ritsema (Peck 2006)
Staphylinidae	Aleocharinae	*Aleodorus partitus* (LeConte) (Hoebeke 1985)
		*Myrmecocephalus gatineauensis* Hoebeke (Hoebeke 1985)
	Micropeplinae	*Micropeplus browni* Campbell (Campbell 1968)
	Oxytelinae	*Coprophilus castoris* Campbell (Campbell 1979)
	Staphylininae	*Gabrius vindex* Smetana (Smetana 1995)
		*Hemiquedius castoris* Brunke & Smetana, sp. n.
		*Quedius campbelli* Smetana (Smetana 1971a)

## Supplementary Material

XML Treatment for Hemiquedius

XML Treatment for Hemiquedius ferox

XML Treatment for Hemiquedius castoris

XML Treatment for Hemiquedius infinitus

## References

[B1] Banfield AWF (1974) The Mammals of Canada. University of Toronto Press, Toronto, 438 pp.

[B2] Bousquet Y (1985) Description of the larva of *Atranus pubescens* (Dejean) (Coleoptera: Carabidae). The Coleopterists Bulletin 39: 329–334.

[B3] Bousquet Y (2012) Catalog of Geadephaga (Coleoptera, Adephaga) of America, north of Mexico. ZooKeys 245: 1–1722. https://doi.org/10.3897/zookeys.245.3416 10.3897/zookeys.245.3416 PMC3577090 23431087

[B4] Brunke A Solodovnikov A (2014) A revision of the Neotropical species of *Bolitogyrus* Chevrolat, a geographically disjunct lineage of Staphylinini (Coleoptera, Staphylinidae). ZooKeys 423: 1–113. https://doi.org/10.3897/zookeys.423.7536 10.3897/zookeys.423.7536 PMC4106094 25061393

[B5] Brunke AJ Chatzimanolis S Schillhammer H Solodovnikov A (2016) Early evolution of the hyperdiverse rove beetle tribe Staphylinini (Coleoptera: Staphylinidae: Staphylininae) and a revision of its higher classification. Cladistics 32: 427–451. https://doi.org/10.1111/cla.12139 10.1111/cla.12139 34740302

[B6] Campbell JM (1968) A revision of the New World Micropeplinae (Coleoptera: Staphylinidae) with a rearrangement of the World species. The Canadian Entomologist 100: 225–267. https://doi.org/10.4039/Ent100225-3

[B7] Campbell JM (1979) *Coprophilus castoris*, a new species of Staphylinidae (Coleoptera) from beaver lodges in eastern Canada. The Coleopterists Bulletin 33: 223–228.

[B8] Chatzimanolis S Cohen IM Schomann AS Solodovnikov A (2010) Molecular phylogeny of the mega-diverse rove beetle tribe Staphylinini (Insecta, Coleoptera, Staphylinidae). Zoologica Scripta 39: 436–449. https://doi.org/10.1111/j.1463-6409.2010.00438.x

[B9] Grebennikov V Jendek E Smirnov ME (2017) Diagnostic and phylogenetic utility of the first DNA barcode library for longhorn beetles (Coleoptera: Cerambycidae) from the Russian Far East. Zootaxa 4276: 441–445. https://doi.org/10.11646/zootaxa.4276.3.9

[B10] Goulet H Bousquet Y (1983) Description of a new *Pterostichus* (Coleoptera: Carabidae) from beaver houses in eastern North America. The Canadian Entomologist 115: 281–286. https://doi.org/10.4039/Ent115281-3

[B11] Hendrich L Moriniere J Haszprunar G Hebert PDN Hausmann A Kohler F Balke M (2014) A comprehensive DNA barcode database for Central European beetles with a focus on Germany: Adding more than 3,500 identified species to BOLD. Molecular Ecology Resources 15: 795–818. https://doi.org/10.1111/1755-0998.12354 25469559 10.1111/1755-0998.12354

[B12] Hoebeke ER (1985) A Revision of the Rove Beetle Tribe Falagriini of America North of Mexico (Coleoptera: Staphylinidae: Aleocharinae). Journal of the New York Entomological Society 93: 913–1018.

[B13] Hoebeke ER (1995) *Coprophilus striatulus* (Coleoptera: Staphylinidae): confirmation of establishment of a Palaearctic oxyteline rove beetle in North America. Entomological News 106: 1–5.

[B14] Larochelle A Lariviere M-C (2003) A Natural History of the Ground-beetles (Coleoptera: Carabidae) of America north of Mexico. Pensoft, Sofia, Bulgaria, 584 pp.

[B15] LeConte J.L. in Schwarz EA (1878) Coleoptera of Florida. Proceedings of the American Philosophical Society 17: 353–472.

[B16] Peck SB (2006) Distribution and biology of the ectoparasitic beaver beetle *Platypsyllus castoris* Ritsema in North America (Coleoptera: Leiodidae: Platypsyllinae). Insecta Mundi 20: 85–94.

[B17] Peck SB (2007) Distribution and biology of the ectoparasitic beetles *Leptinillus validus* (Horn) and *L. aplodontiae* Ferris of North America (Coleoptera: Leiodidae: Platypsyllinae). Insecta Mundi 55: 1–7.

[B18] Ratnasingham S Hebert PDN (2013) A DNA-Based Registry for All Animal Species: The Barcode Index Number (BIN) System. PLoS ONE 8(8): e66213. https://doi.org/10.1371/journal.pone.0066213 10.1371/journal.pone.0066213 PMC3704603 23861743

[B19] Rosell F Bozsér O Collen P Parker H (2005) Ecological impact of beavers *Castor fiber* and *Castor canadensis* and their ability to modify ecosystem. Mammal Revues 35: 248–276. https://doi.org/10.1111/j.1365-2907.2005.00067.x

[B20] Shorthouse DP (2010) SimpleMappr, an online tool to produce publication-quality point maps. [Retrieved from http://www.simplemappr.net. Accessed July 1, 2017]

[B21] Smetana A (1971a) Revision of the tribe Quediini of North America north of Mexico (Coleoptera: Staphylinidae). Memoirs of the Entomological Society of Canada No. 79: 1–303. https://doi.org/10.4039/entm10379fv

[B22] Smetana A (1971b) Revision of the tribe Quediini of America North of Mexico (Coleoptera: Staphylinidae). Supplementum 1. The Canadian Entomologist 103: 1833–1848. https://doi.org/10.4039/Ent1031833-12

[B23] Smetana A (1978) Revision of the tribe Quediini of America North of Mexico (Coleoptera: Staphylinidae). Supplementum 4. The Canadian Entomologist 110: 815–840. https://doi.org/10.4039/Ent110815-8

[B24] Smetana A (1990) Revision of the tribe Quediini of America North of Mexico (Coleoptera: Staphylinidae). Supplementum 6. The Coleopterists Bulletin 44: 95–104.

[B25] Smetana A (1995) Rove beetles of the subtribe Philonthina of America north of Mexico (Coleoptera: Staphylinidae). Classification, phylogeny and taxonomic revision. Memoirs on Entomology, International 3: 1–945.

[B26] von Beeren C Maruyama M Kronauer DJC (2016) Community Sampling and Integrative Taxonomy Reveal New Species and Host Specificity in the Army Ant-Associated Beetle Genus *Tetradonia* (Coleoptera, Staphylinidae, Aleocharinae). PLoS One 11: e0165056. https://doi.org/10.1371/journal.pone.0165056 10.1371/journal.pone.0165056 PMC5102370 27829037

[B27] Webster R DeMerchant I (2012a) New Coleoptera records from New Brunswick, Canada: Gyrinidae, Carabidae, and Dytiscidae. ZooKeys 179: 1–10. https://doi.org/10.3897/zookeys.179.2582 10.3897/zookeys.179.2582 PMC3337048 22539881

[B28] Webster R DeMerchant I (2012b) New Staphylinidae (Coleoptera) records with new collection data from New Brunswick, Canada: Paederinae. ZooKeys 186: 273–292. https://doi.org/10.3897/zookeys.186.2504 10.3897/zookeys.186.2504 PMC3349198 22577324

